# Contributions of chaperone and glycosyltransferase activities of *O*-fucosyltransferase 1 to Notch signaling

**DOI:** 10.1186/1741-7007-6-1

**Published:** 2008-01-14

**Authors:** Tetsuya Okajima, BVVG Reddy, Tsukasa Matsuda, Kenneth D Irvine

**Affiliations:** 1 Nagoya University Graduate School of Bioagricultural Sciences, Department of Applied Molecular Biosciences, Furo-cho, Chikusa-ku, Nagoya 464-8601, Japan; 2 Howard Hughes Medical Institute, Waksman Institute and Department of Molecular Biology and Biochemistry, Rutgers The State University of New Jersey, Piscataway, NJ 08854, USA

## Abstract

**Background:**

*O*-fucosyltransferase1 (OFUT1) is a conserved ER protein essential for Notch signaling. OFUT1 glycosylates EGF domains, which can then be further modified by the *N*-acetylglucosaminyltransferase Fringe. OFUT1 also possesses a chaperone activity that promotes the folding and secretion of Notch. Here, we investigate the respective contributions of these activities to Notch signaling in *Drosophila*.

**Results:**

We show that expression of an isoform lacking fucosyltransferase activity, *Ofut1*^*R*245*A*^, rescues the requirement for *Ofut1 *in embryonic neurogenesis. Lack of requirement for *O*-fucosylation is further supported by the absence of embryonic phenotypes in *Gmd *mutants, which lack all forms of fucosylation. Requirements for *O*-fucose during imaginal development were evaluated by characterizing clones of cells expressing only *Ofut1*^*R*245*A*^. These clones phenocopy *fringe *mutant clones, indicating that the absence of *O*-fucose is functionally equivalent to the absence of elongated *O*-fucose.

**Conclusion:**

Our results establish that Notch does not need to be *O*-fucosylated for *fringe*-independent Notch signaling in *Drosophila*; the chaperone activity of OFUT1 is sufficient for the generation of functional Notch.

## Background

Notch proteins are receptors for a conserved intercellular signaling pathway that mediates a wide variety of cell-fate decisions during animal development [1]. Notch activity needs to be regulated precisely, and aberrant Notch activity is associated with a number of human diseases including cancers and congenital syndromes.

Notch signaling is influenced by two conserved glycosyltransferases, *O*-fucosyltransferase1 (OFUT1) and Fringe (FNG) [2]. FNG transfers *N*-acetylglucosamine (GlcNAc) in a β1,3 linkage onto *O*-linked fucose on EGF domains [3,4]. Fringe was first identified because of its role in modulating Notch signaling during the development of the *Drosophila *wing, where it both potentiates the activation of Notch by one ligand, Delta, and inhibits the activation of Notch by another ligand, Serrate [5]. These opposing effects of Fringe on the activation of Notch by its ligands, together with the restriction of normal Fringe expression to dorsal wing cells, help to position a stripe of Notch activation along the dorsal-ventral (D-V) compartment boundary. This stripe of Notch activation is then essential for the further growth and patterning of the wing. Fringe also helps to regulate Notch activation in other *Drosophila *tissues; however, there are many Notch signaling events in *Drosophila*, such as the role of Notch in limiting the number of neural precursor cells (lateral inhibition) that are Fringe-independent. Similarly, vertebrate Fringe proteins are important regulators of Notch signaling in some contexts, but not in others [1,2].

OFUT1 catalyzes the transfer of fucose from GDP-fucose, the universal donor for fucosyltransferases, onto EGF domains [6]. It thus generates the *O*-linked fucose that is the substrate for FNG. Genetic studies of *Ofut1 *in flies, and its homolog *Pofut1 *in mice, have indicated that it has a much broader role in Notch signaling than FNG, and indeed appears to be universally required for all Notch signaling [7-9]. While this was initially taken to reflect a universal requirement for *O*-fucose on Notch, more recently a second function for OFUT1 was identified [10]. OFUT1 is a soluble ER protein [10,11] and, at least in *Drosophila*, acts as a Notch chaperone, facilitating the folding and secretion of Notch[10].

The fact that OFUT1 possesses both fucosyltransferase and chaperone activity for Notch raises the question of the respective contributions of these two activities to the genetic requirement for OFUT1 in Notch signaling. In this study, we have addressed this by examining the *in vivo *activity of Notch produced in cells with OFUT1 chaperone activity, but lacking fucosyltransferase activity. Surprisingly, we find that Notch expressed by these cells is a functional receptor. Our observations indicate that *O*-fucosylation is dispensable for many Notch signaling events during *Drosophila *development.

## Results

### *O*-fucosylation of Notch is not required during embryonic neurogenesis

**Figure 1:**
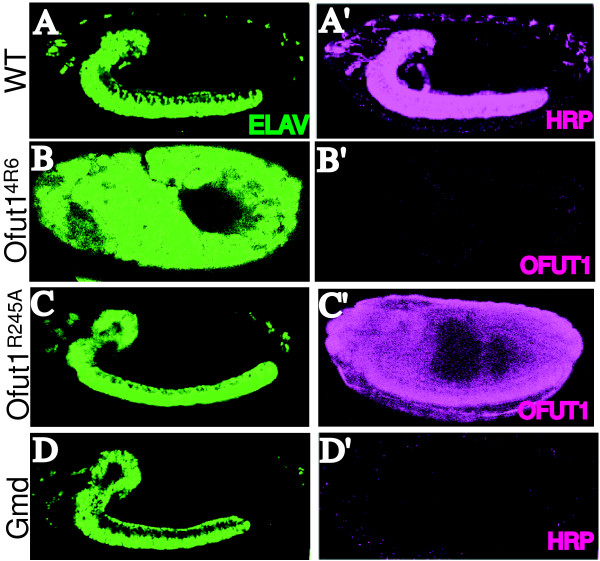
***O*-fucose is not required during embryonic neurogenesis**. Embryos immunostained with anti-ELAV (green) and either anti-HRP or anti-OFUT1 (magenta) antibodies, as indicated. Panels marked prime show separate channels of the same embryo. (A)Wild-type embryo. (B) *Ofut1*^4*R*6 ^mutant embryo from *Ofut1*^4*R*6 ^germline clone. A neurogenic phenotype is revealed by the expansion of ELAV staining. (C) *Ofut1*^4*R*6 ^mutant embryo from *Ofut1*^4*R*6 ^germline clone expressing *Ofut1*^*R*245*A *^from a genomic rescue construct(*Ofut1*^*R*245*A*^*[18.1]*). OFUT1^R245A ^expression is visible. ELAV staining shows the absence of a neurogenic phenotype. (D) *Gmd*^1 ^mutant embryo derived from *Gmd*^1 ^germline clone. Absence of fucosylation was confirmed by the absence of the HRP epitope; neurogenesis appears normal.

We have taken two complementary approaches to evaluate the respective contributions of the fucosyltransferase and chaperone activities of OFUT1 to Notch signaling. First, we employed a mutant isoform of OFUT1, OFUT1^R245A^. This mutation alters an invariant arginine within the putative GDP binding site, and it eliminates detectable fucosyltransferase activity, while retaining chaperone activity [10]. A genomic rescue construct carrying the *Ofut1*^*R*245*A *^mutation was created, and then introduced into flies by P element-mediated transformation. The ability of the OFUT1^R245A ^isoform to rescue Notch signaling phenotypes was then assayed in animals lacking wild-type OFUT1. As *Ofut1 *is provided maternally to embryos, this was done by making germline clones with a null allele of *Ofut1*, *Ofut1*^4*R*6^, in animals containing the *Ofut1*^*R*245*A *^genomic construct inserted on the same chromosome arm. In the absence of any rescue construct, animals lacking maternal and zygotic *Ofut1 *exhibit a strong neurogenic phenotype, in which impairment of lateral inhibition causes excess neurons to be produced at the expense of epidermal cells [9]. This phenotype is characteristic of mutations in Notch pathway genes and can be visualized by staining with antibodies against a marker for neuronal cells, ELAV (Figure 1B, cf. Figure 1A). Significantly, when *Ofut1*^*R*245*A *^is provided to *Ofut1*^4*R*6 ^germline clones embryos, embryonic neurogenesis, as revealed by ELAV staining, appears indistinguishable from that in wild-type embryos (Figure 1C). These embryos lacking O-FucT activity can hatch, but die as first instar larvae. This result implies that the fucosyltransferase activity of OFUT1 is not essential for Notch signaling during lateral inhibition.

As an independent test of the requirement for Notch fucosylation, we analyzed a mutant in which wild-type OFUT1 is present but unable to fucosylate Notch owing to the absence of its donor substrate, GDP-fucose. GDP-fucose is generated in the cytoplasm from GDP-mannose by enzymes including GDP-D-mannose dehydratase (GMD). Since *Drosophila *lack a salvage pathway for GDP-fucose synthesis [12], and GDP-fucose is the common donor substrate for all fucosyltransferases, animals lacking GMD are expected to be unable to effect all types of fucosylation. Animals that are mutant for a null allele of *Gmd*, *Gmd*^1 ^[10], die as larvae with no neurogenic phenotype (not shown), but the survival of *Gmd *zygotic mutants could have been a result of maternally provided product. Thus, requirements for *Gmd *during embryonic development were evaluated by making germline clones. Confirmation of the general deficit in fucosylation in these embryos was provided by staining with anti-HRP antibodies, which recognize a fucose-containing epitope on neuronal *N*-glycans [13]. Anti-HRP staining was completely eliminated in animals lacking both maternal and zyogotic contributions of *Gmd *(Figure 1D). Strikingly, however, Notch signaling in these embryos, as visualized by ELAV staining, was indistinguishable from wild type (Figure 1D) and *Gmd *germline clone embryos are able to complete embryogenesis and hatch before dying as first instar larvae. These results establish that all fucose modifications are dispensable for lateral inhibition, as well as all other developmental processes essential for the hatching of a *Drosophila *embryo.

### Role of Notch *O*-fucosylation in the wing imaginal disk

**Figure 2:**
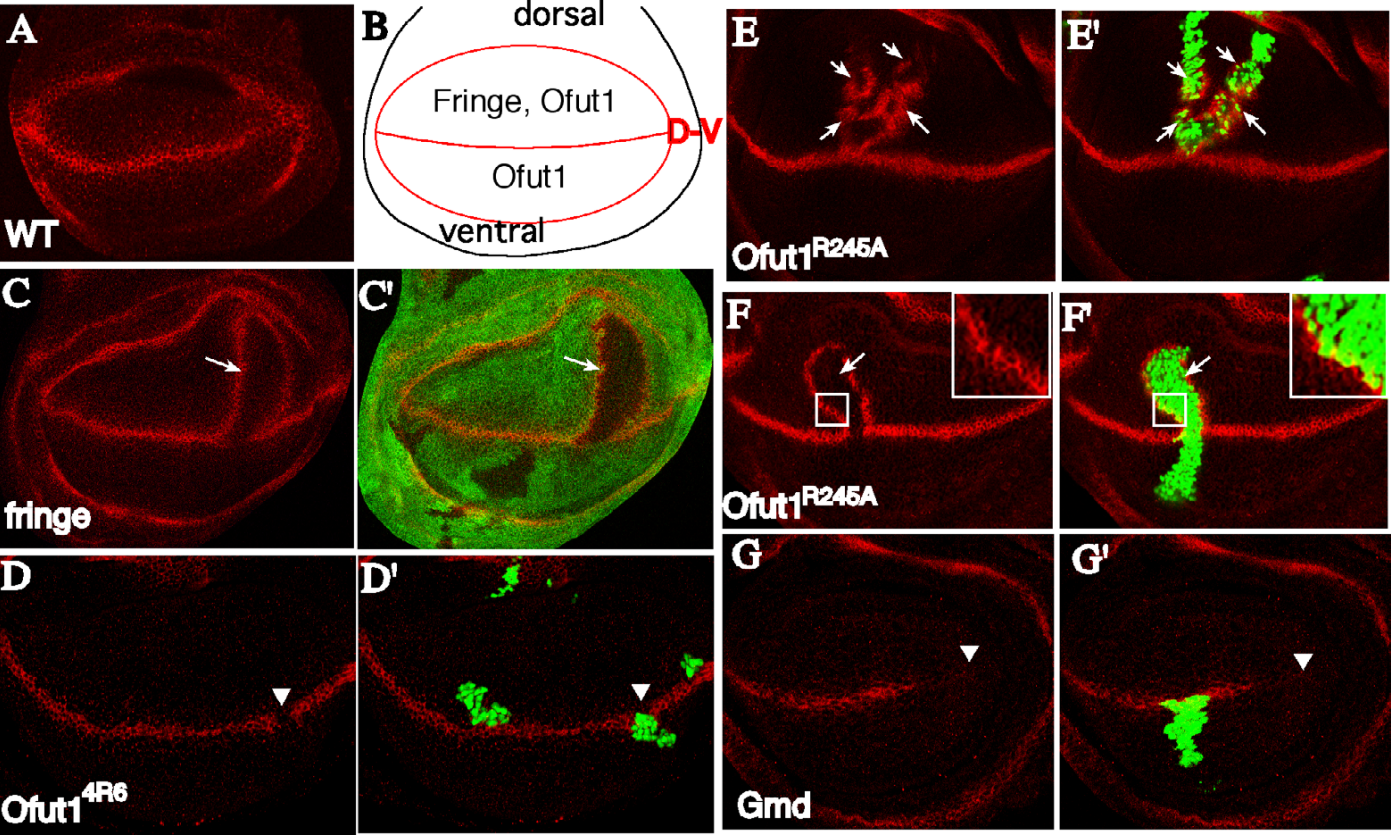
***O*-fucose is not required during Notch signaling in wing disks**. Third instar wing disks stained for WG expression (red), with dorsal up and anterior to the left. (A)Wild-type. (B) Schematic drawing. WG expression is indicated in red. D-V indicates dorsal-ventral boundary. *Ofut1 *is expressed in both compartments whereas *fng *is expressed only dorsally. (C) *fng *mutant clones, marked by absence of GFP (green). Ectopic WG is indicated (arrow). (D) *Ofut1*^4*R*6 ^mutant clones, marked by presence of GFP (green, using the MARCM technique [16]). Loss of WG is indicated (arrowhead). (E), (F) Rescue experiments, with clones positively marked by GFP (green) using MARCM. OFUT1^R245A ^was expressed in *Ofut1*^4*R*6 ^clones either by expressing a *UAS-Ofut1*^*R*245*A*^*[28.3] *construct under *tubulin*-Gal4 control (E) or employing the *Ofut1*^*R*245*A*^*[23.1] *genomic construct recombined onto an *Ofut1*^4*R*6 ^chromosome (F). Ectopic WG is indicated (arrow). OFUT1 expression was confirmed by anti-OFUT1 staining (not shown). The inset depicts a high magnification image of the boxed area with WG expression inside (yellow) and outside (red) of the clone border evident. (G) *Gmd*^1 ^clones. Large clones mutant for *Gmd*^1 ^(marked by loss of GFP), occupying nearly the entire disk, were generated using the *Minute *technique. WG expression appears normal in *Gmd*^1 ^cells surrounding the *Gmd*^+ ^cells, but further away loss of WG expression is evident (arrowhead).

Notch signaling participates in many different processes throughout development and many of the factors that modulate Notch signaling are context specific. For example, *fng *is dispensable for Notch functions during embryonic neurogenesis, but plays a critical role in the developing wing. In the wing, *fng *is expressed by dorsal cells and its positive and negative effects on signaling by Delta and Ser, respectively, position a stripe of Notch activation along the edge of *fng *expression at the D-V boundary [5]. The critical role of *fng *in positioning Notch activation is evidenced by the observation that establishing novel *fng *expression boundaries by creating *fng *mutant clones in dorsal cells results in the induction of ectopic stripes of Notch activation, which can be visualized by examining expression of targets of Notch signaling, such as Wingless (WG; see Figure 2C) [14]. In contrast, *Ofut1 *mutant clones are associated with a complete loss of Notch activation in the wing (Figure 2D) [9,15], equivalent to that observed in *Notch *mutant clones.

To evaluate requirements for Notch *O*-fucosylation during wing development, we again took advantage of the ability to rescue the chaperone, but not the fucosyltransferase, activities of OFUT1 by expression of *Ofut1*^*R*245*A*^. *Ofut1*^*R*245*A *^was expressed in *Ofut1*^4*R*6 ^null mutant clones using two different methods. In one set of experiments, we expressed *Ofut1*^*R*245*A *^from a genomic rescue construct, such that *Ofut1*^*R*245*A *^was expressed under its own promoter. In an alternative approach, *Ofut1*^*R*245*A *^was over-expressed in *Ofut1*^4*R*6 ^mutant clones using the mosaic analysis with a repressible cell marker (MARCM) technique [16], which employs the UAS-Gal4 system to drive expression under the control of a heterologous promoter. Both methods yielded similar results: when *Ofut1*^*R*245*A *^expression is similar to endogenous *Ofut1 *levels, the rescued clones phenocopy *fng *mutant clones (Figures 2E and 2F, cf. Figure 2C). Thus, where the clones intersected the normal D-V boundary, normal WG expression is lost, whereas the borders of clones within the dorsal compartment can be associated with ectopic WG expression. This striking phenotype has two important implications. First, because WG expression can be induced within mutant cells expressing only *Ofut1*^*R*245*A*^, it indicates that, as during embryonic neurogenesis, modification of Notch with *O*-fucose is not required for it to function as a receptor. Second, it indicates that the absence of *O*-fucose is functionally equivalent to the absence of elongated (i.e. FNG-modified) *O*-fucose in the developing wing.

We also attempted to extend these observations by examining *Gmd *mutants. Animals that are mutant for a null allele, *Gmd*^1^, exhibit decreased growth and loss of WG expression in the wing imaginal disks, consistent with a deficit in Notch signaling [10]. To investigate whether this phenotype reflects a specific requirement for *Gmd *in FNG-dependent Notch signaling, or a more general requirement for *Gmd *in Notch signaling, we created clones of cells homozygous for *Gmd*^1^. However, in most cases these did not show obvious *Notch*-loss-of-function phenotypes (not shown). Only when the *Minute *technique was used to generate disks in which all, or almost all, of the wing was composed of mutant tissue was a loss of WG expression observed, and in all cases the loss of WG expression was non-autonomous (Figure 2G). A non-autonomous phenotype in clones has been reported previously for a mutation in UDP-glucose dehydrogenase, which is required for the synthesis of heparan sulfate [17]. We suggest that a general non-autonomy of mutations in genes that participate in nucleotide sugar biosynthesis could be explained if nucleotide sugars can diffuse through cells via gap junctions. Owing this non-autonomy, *Gmd *clones could not be directly compared with *fng *or *Ofut1 *clones.

### Over-expression of OFUT1 can inhibit Notch signaling independently of its fucosyltransferase activity

**Figure 3:**
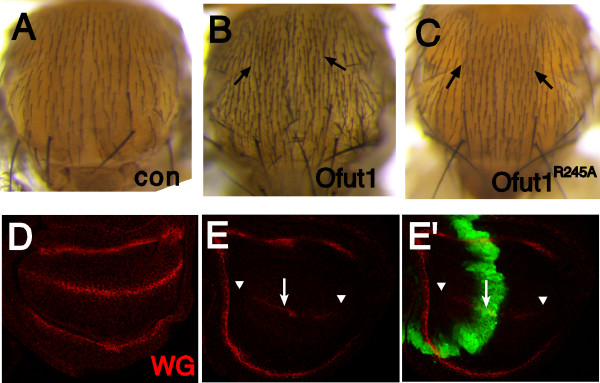
**Over-expression of OFUT1 inhibits Notch activity non-enzymatically and non-autonomously**. Adult nota from (A) *ap-Gal4 *control, (B) *ap-Gal4; UAS-Ofut1 [8.2]*and (C) *ap-Gal4; UAS-Ofut1*^*R*245*A*^*[28.3]*. Note the increased density of bristles (arrows) when OFUT1 is over-expressed. All of the crosses are performed at 25°C. The average number of microchaetes present in the acrostichal region of the notum are as follows: (A) 97 ± 10, *n *= 3; (B) 147 ± 25, *n *= 3; (C) 140 ± 15, *n *= 4. (D) WG expression (red) in a wild-type wing disk. (E) WG expression (red) in a *ptc-Gal4 UAS-GFP; UAS-Ofut1 [11.1] *wing disk. WG expression along the D-V boundary is decreased both inside (arrow) and outside (arrowhead) of the *ptc *stripe (green).

Loss of *Ofut1 *impairs Notch signaling, but over-expression of *Ofut1 *can also impair Notch signaling [7]. This observation led to the suggestion that high levels of Notch *O*-fucosylation might suppress Notch signaling. However, the determination that it is the chaperone activity, rather than the fucosyltransferase activity, that is universally required for Notch signaling prompted us to re-examine the basis for the inhibition of Notch signaling associated with OFUT1 over-expression. Towards that end, *Ofut1*^*R*245*A *^was expressed at high levels in the developing notum under the control of *ap-Gal4*. Expression of wild-type *Ofut1 *(*UAS-Ofut1 [8.2]*) under *ap-Gal4 *control interferes with Notch-mediated lateral inhibition, and so results in the production of extra bristles (Figure 3B, cf. Figure 3A) [7]. The effect on bristles was only evident in microchaetes, but not in macrochaetes. Over-expression of *Ofut1*^*R*245*A *^(*UAS-Ofut1*^*R*245*A*^*[28.3]*) under *ap-Gal4 *control results in a similar phenotype (Figure 3C). This observation, together with another recent study [18], indicate that the over-expression phenotype is independent of OFUT1's fucosyltransferase activity. Interestingly, when much stronger *Ofut1 *expression (relative to Figure 3B) was induced in a thin stripe of cells along the anterior-posterior (A-P) compartment boundary using the *ptc-Gal4 *driver with *UAS-Ofut1 [11.1]*, WG expression was inhibited non-autonomously (Figure 3E, cf. Figure 3D). As extracellular concentrations of nucleotide sugars are generally thought to be too low to support glycosylation, this non-autonomous affect is also consistent with a fucosylation-independent activity.

### OFUT1 is required for secretion of Notch from the ER

The observation that the fucosyltransferase activity of OFUT1 is not required for all Notch signaling events implies that the previously described Notch chaperone activity of OFUT1 is its most critical function. Recently, however, our identification of this chaperone activity has been questioned and two additional activities for OFUT1 have been proposed in its place: the first, independent of its fucosyltransferase activity, promotes endocytosis of Notch from the plasma membrane to the early endosome [18]; the second, dependent upon fucosyltransferase activity, promotes trafficking of Notch from the plasma membrane to the sub-apical complex and adherens junctions [18]. The two most critical experiments distinguishing between the chaperone model and these trafficking models for OFUT1 function relate to the localization of Notch in cells lacking OFUT1.

**Figure 4:**
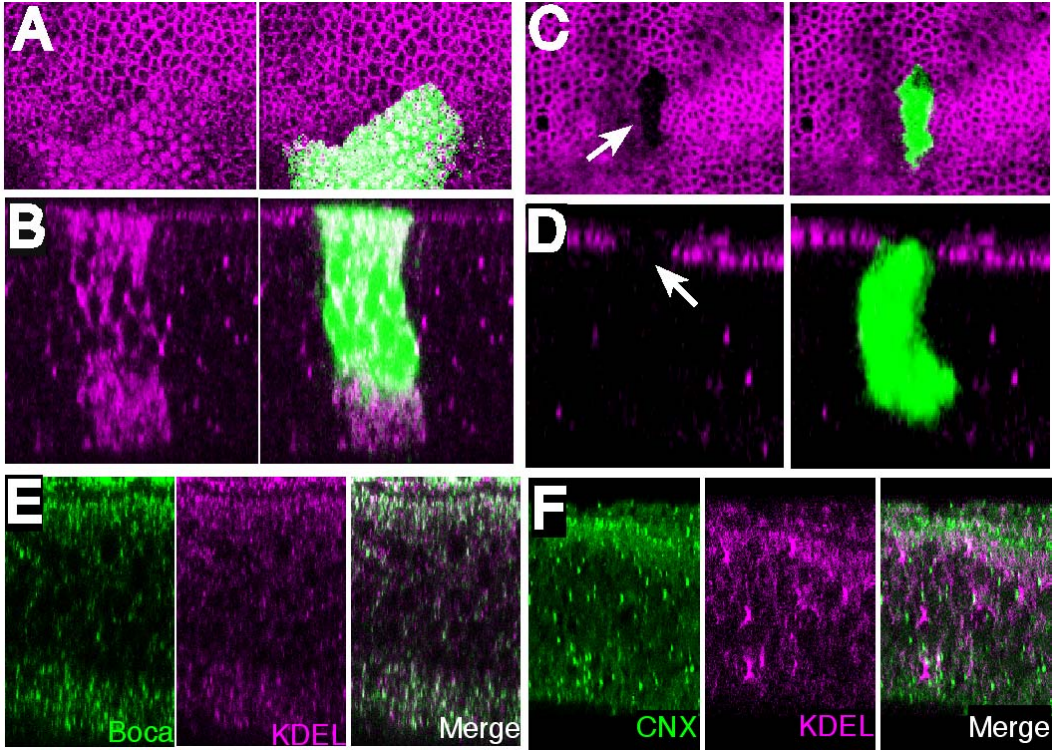
**Notch does not reach the cell surface in *Ofut1 *mutant cells**. (A)-(D) Wing disks with *Ofut1 *mutant clones (green), stained with antibodies against the Notch extracellular domain (magenta). (A) Horizontal and (B) vertical sections of a disk stained after detergent treatment. Apical is up. Increased and mis-localized Notch protein is observed within *Ofut1 *mutant cells (green), as reported previously using antibodies against the intracellular domain [10]. (C) Horizontal and (D) vertical sections of a disk stained without detergent treatment. An *Ofut1 *mutant clone is devoid of cell surface Notch (arrow), but Notch is readily detected along the surface of wild-type cells. (E),(F) Correlations in localization among ER markers. Vertical sections of a wing disk doubly stained with KDEL (magenta) and with either (E) Boca or (F) Calnexin (green). Additional examples are shown in additional file 1.

First, it was claimed that in contrast to our report that Notch is not secreted to the cell surface in the absence of OFUT1 [10], Notch is secreted to the plasma membrane in *Ofut1 *mutant cells [18]. This claim was based on an assay in which anti-Notch antibodies were added to live disks. However, this is not an effective assay for cell surface localization, because a cell surface receptor is not required for bulk endocytosis (e.g. even fluorescent dextran is efficiently endocytosed by disk cells [19]), and once endocytosed, antibodies could be spread throughout the secretory pathway and then accumulate wherever there are significant epitope concentrations. A standard assay for cell surface localization is to determine whether the accessibility of epitopes requires membrane permeabilization. Thus, we performed immunostaining using antibodies directed against the extracellular domain of Notch, both in the presence and in the absence of detergent. When wing disk cells are permeabilized with detergent, wild-type cells exhibit both intracellular staining as well as apical hexagonal staining, corresponding to the normal cell surface localization of Notch near the adherens junctions (Figures 4A and 4B). *Ofut1 *mutant cells show increased Notch staining (Figures 4A and 4B), which we have previously shown overlaps with ER markers [10] (see Additional file 1). To demonstrate that the Notch accumulation near the apical surface in *Ofut1 *mutant cells is not exposed on the cell surface, we stained disks without detergent treatment. Under these conditions, no staining was observed in *Ofut1 *mutant cells, whereas neighboring wild-type cells exhibit normal apical surface staining (Figures 4C and 4D). These results are consistent with previous data using *Drosophila *S2 cells [10] and reconfirm that OFUT1 is required for secretion of Notch to the cell surface in wing disk cells.

The second critical piece of evidence presented against the chaperone model was a claim that the Notch that accumulates in *Ofut1 *mutant cells is not actually in the ER. This claim relies on the belief that the ER is homogeneous organelle, that is, that all ER proteins are homogeneously distributed. Both our studies [10] and those of Sasaki *et al.*[18] reveal overlap between Notch and ER markers in *Ofut1 *mutant or RNAi-depleted cells. At the same time, we agree that Notch does not overlap perfectly with ER markers in all focal planes; indeed, it is for this reason that we originally thought that Notch was not in the ER in the absence of OFUT1 [7]. However, we now think that differences between Notch and other markers reflect ER heterogeneity. In support of this idea, we have investigated the relative distributions of five different ER markers in wing imaginal disks cells: two dedicated ER chaperones (OFUT1 and Boca), a classic ER protein marker (Calnexin), a synthetic ER protein (GFP with a KDEL ER retention signal added) and bulk ER proteins (using an anti-KDEL antibody). Each of these ER markers exhibits partial, but not perfect, overlap with other ER markers (see Figures 4E and 4F and Additional file 1) [10]. This observation of ER heterogeneity emphasizes that the lack of perfect correspondence among markers cannot be taken as compelling evidence for the absence of ER localization of Notch in *Ofut1 *mutant cells. Together with observations that the accumulated Notch in *Ofut1 *cells does not overlap markers for any other vesicles or organelles [10,18], that the distribution of Notch in *Ofut1 *mutant cells is similar to the distribution of OFUT1 itself [10], which by several criteria is an ER protein [10,11], and that Notch does partially overlap with a variety of ER markers in *Ofut1 *mutant or depleted cells [10,18], these observations are consistent with the conclusion that Notch accumulation within *Ofut1 *mutant cells is in the ER.

## Discussion

The observations we describe here lead to the unexpected conclusion that the fucosyltransferase activity of OFUT1 is not essential for all Notch signaling in *Drosophila*. The lack of requirement for Notch *O*-fucosylation is evidenced by the observation that expression of a form of OFUT1 that provides chaperone activity, but lacks fucosyltransferase activity, is sufficient to enable Notch receptor activation in at least two different contexts: during embryonic neurogenesis, where Notch signaling effects lateral inhibition, and during wing disk development, where inductive Notch signaling establishes specialized D-V boundary cells. Indeed, because *Ofut1*^*R*245*A*^-rescued animals complete embryogenesis and hatch without grossly evident abnormalities, it is likely that many other Notch signaling events also do not require Notch fucosylation. Although reagents that would enable direct visualization of the *O*-fucosylation state of Notch in cells expressing *Ofut1*^*R*245*A *^do not exist, prior characterization of this mutant isoform indicates that it is completely defective in fucosylation [10]. Further, the observation of *fng*-like phenotypes in the wing provides a genetic argument that fucosylation is lacking in cells expressing only *Ofut1*^*R*245*A*^, as if *O*-fucose were present on Notch then it should be modified by FNG. Moreover, in a completely independent approach, the lack of requirement for Notch fucosylation was demonstrated by the observation of normal neurogenesis and completion of embryogenesis in *Gmd *mutants, whose general deficit in fucosylation was confirmed by anti-HRP staining. The absence of *O*-fucosylation in *Gmd *mutants is also consistent with the prior observation that Notch signaling at the D-V boundary of the wing is lost in *Gmd *mutants [10], as this is a *fng*-dependent process. Collectively, these results indicate that Notch receptors can transduce signals without modification by *O*-fucose. Prior results suggesting that *O*-fucose is required for Notch-ligand binding [9,15] can be explained by the requirement for the chaperone activity of OFUT1. Indeed, a bacterially expressed fragment of mammalian Notch1 can bind to Delta in spite of the absence of glycosylation [20].

The observation that *O*-fucose is not universally required for Notch signaling does not mean that it has no effect. In acting as a substrate for FNG, *O*-fucose clearly has an important modulatory role. It is, however, conceivable that this is the only absolute requirement for *O*-fucose, as genetic studies of the *Ofut1*^*R*245*A *^allele, as well as genetic studies of *Gmd*, are consistent with the possibility that all requirements for *O*-fucose might be explained by its role in FNG-dependent modulation of Notch: like *Ofut1*^*R*245*A*^-rescued animals, or *Gmd *maternal and zygotic mutant animals, *fng *mutant animals die as first instar larvae, without obvious affects on embryonic neurogenesis [21]. The apparently normal embryogenesis of *Gmd *mutants is particularly striking, as fucose is a common component of insect *N*-glycans.

The similarity between *Ofut1*^*R*245*A*^-rescued clones and *fng *mutant clones in the wing is also informative in terms of the nature of the requirement for FNG modification. That is, the observation that loss of *O*-fucose results in the same phenotype as loss of *O*-fucose elongation implies that modulation of Notch signaling by FNG is affected because a specific glycan created as a consequence of FNG modification (i.e. elongated *O*-fucose) alters Notch-ligand interactions, rather than because elongation of *O*-fucose covers up a glycan (i.e. the *O*-fucose monosaccharide) that promotes Serrate-Notch interactions or impairs Delta-Notch interactions.

Although the phenotypes of *Ofut1*^R245A ^and *Gmd *might be explained simply by the requirement for FNG-dependent elongation, there are nonetheless some indications that the monosaccharide form of *O*-fucose can have an influence on Notch. *In vitro *binding revealed that elimination of the *O*-fucose modification site located in the ligand-binding region (EGF repeat 12) of *Drosophila *Notch causes elevated Serrate binding in the absence of Fringe [22]. Mutagenesis of the *O*-fucose site on EGF12 of Notch in mammalian cells also influenced Notch signaling, although in this case the result was an impairment of Delta-like 1 or Jagged-1 activation of Notch1 in cultured cells [23], and impaired Notch signaling *in vivo *(Ge and Stanley, personal communication). Moreover, the introduction of a novel *O*-fucosylation site on EGF repeat 14, as observed in the *N*^*spl *^mutation, causes ectopic Notch activation during eye development, independently of Fringe activity [24]. These observations are, however, subject to the caveat that the affects of these mutations might not actually be a result of their effects on *O*-fucosylation. Cell-based ligand binding assays have revealed that a soluble form of Notch produced from cells over-expressing wild-type *Ofut1 *results in increased binding to Serrate but decreased binding to Delta [15], whereas Notch from *Ofut1*^*R*245*A *^over-expressing cells exhibits increased binding to both Serrate and Delta [10]. These differing affects of OFUT1 versus OFUT1^R245A ^on Delta-binding suggest that increased *O*-fucosylation can modulate Notch-Delta interactions. A study of a *Gmd*-deficient mammalian cell line, Lec13, revealed that Jagged-1 dependent activation of Notch1 was reduced in the mutant cells, suggesting that *O*-fucose positively regulates Notch1 activation by Jagged-1 in this context [3,25]. Finally, we note that the expression of *Ofut1 *is developmentally regulated in *Drosophila*, exhibiting a complex spatial and temporal pattern [7]. Thus, while *O*-fucose is clearly not essential for all Notch signaling, it might still have a modulatory role in some contexts.

Our studies have focused on the fucosyltransferase and the chaperone activities of OFUT1 as distinct functions, genetically separable by the R245A mutation. Nonetheless, from an evolutionary perspective it would be surprising if these activities were unrelated. Given that OFUT1 acts during the folding process and dissociates from EGF domains upon *O*-fucosylation, it is tempting to speculate that *O*-fucose might serve as a tag for correctly folded EGF domains, thus directing OFUT1 to incompletely folded EGF domains. Therefore, although *O*-fucosylation is not absolutely required for Notch receptor activity, it might still affect the efficiency of the chaperone activity of OFUT1. In support of this hypothesis, we have observed modest decreases in Notch secretion in S2 cells treated with double-stranded RNA corresponding to *Gmd *(Tetsuya Okajima, unpublished observations).

Our studies also suggest the molecular basis for the previous observation that over-expression of OFUT1 inhibits Notch signaling [7]. We showed that over-expressed OFUT1 inhibits Notch signaling non-autonomously and non-enzymatically (Figure 3). Although OFUT1 is predominantly an ER protein, a small fraction is secreted from cells [7,10], which presents the possibility that the non-autonomous affect of OFUT1 might be effected through direct interaction of secreted OFUT1 with Notch or its ligands, presumably mediated by its ability to associate with EGF domains [6,10]. Indeed it has recently been observed that secreted OFUT1 can promote endocytosis of Notch [26], which could provide an explanation for the inhibition of Notch signaling associated with OFUT1 over-expression. We note, however, that the biological relevance of this phenomenon remains to be determined: it can occur when OFUT1 is over-expressed, but it is not clear whether it is significant at endogenous expression levels. Nonetheless, it is of potential pharmacological interest to note that OFUT1 can act as a soluble inhibitor of Notch signaling.

## Conclusion

In summary, our findings demonstrated for the first time that *O*-fucose modification on Notch receptors is not absolutely required for their activity in *fringe*-independent developmental processes in *Drosophila*, and that successful folding mediated by chaperone activity of OFUT1 is sufficient to generate functional Notch receptors.

## Methods

### Stocks

*Ofut1*^4*R*6 ^was obtained from K Matsuno [9]. *UAS-Ofut1*^*R*245*A*^*[28.3] *and *Gmd*^1 ^are described in [10]. The Gal4 drivers used were *ptc-Gal4 *(Flybase ID number; FBti0002124), *ap-Gal4 *(FBti0002785). *UAS-iOfut1 [16.2]*, *UAS-iOfut1 [12.3], UAS-Ofut1 [11.1] *and *UAS-Ofut1 [8.2] *(insertion on chromosome 2) were obtained as described previously [7]. *UAS-Ofut1 [11.1] *provides stronger expression than *UAS-Ofut1[8.2]*.

### Genetics

To obtain mutants lacking both maternal and zygotic *Gmd*, germline clones of *Gmd*^1 ^were made by crossing *Gmd*^1^*FRT 40A*/*CyO *virgins to *hs flp; ovo*^*D*^*FRT 40A*/*CyO *males and heat shocking the progeny at 38°C for 1 h on two successive days during first and second larval instar. Then, *hs flp*; *Gmd*^1^*FRT40A*/*ovo*^*D*^*FRT 40A *female progeny that contained *Gmd*^1 ^germline clones were crossed to *Gmd*^1^*/CyO; twi-Gal4 UAS-GFP *males.

To obtain mutants lacking both maternal and zygotic *Ofut1*, germline clones of *Ofut1*^4*R*6 ^were made by crossing *FRT42B [G13] Ofut1*^4*R*6^/*CyO *virgins to *hs-flp [122]; FRT42B [G13] ovo*^*D*^/*CyO *males and heat shocking the progeny at 38°C for 1 h on two successive days during the second and early third larval instar. Then, *hs-flp [122]*; *FRT42B [G13] Ofut1*^4*R*6^/*FRT42B [G13] ovo*^*D *^female progeny that contained *Ofut1*^4*R*6 ^germline clones were crossed to *FRT42B [G13] Ofut1*^4*R*6^*/CyO, twi-Gal4 UAS-GFP *males.

To generate larger clone for *Gmd*^1^in disks, the *Minute *technique [27] was employed. *M(2) Ubi-GFP FRT40A/CyO act-GFP *flies were crossed to *yflp; FRT40A Gmd*^1^*/CyO *flies and the progeny were heat shocked at 38°C for 1 h during first larval instar. The homozygous *Gmd*^1 ^cells produced by mitotic recombination lack the *Minute *mutation and, thus, divide more quickly than the surrounding cells heterozygous for *Minute*. The homozygous *Minute *clones cannot survive.

To express *Ofut1*^*R*245*A *^in *Ofut1 *mutant backgrounds, the MARCM technique [27,28] was used. This technique allows the generation of mosaic clones that are mutant for one gene and expressing another gene under the control of a UAS promoter. Mosaic clones were generated by crossing *y w hs-Flp [122] tub-Gal4 UAS-GFP:nls; FRT42B [G13] hsp:Myc tub-Gal80 [LL2]/CyO *females to *FRT42B [G13] Ofut1*^4*R*6^*/CyO*; *UAS-Ofut1*^*R*245*A *^*[28.3]/TM6B *males. The expression of *Ofut1*^*R*245*A *^is suppressed by the ubiquitous expression of the Gal80 transcriptional repressor. However, FRT-mediated mitotic recombination generating mutant clones for *Ofut1*^4*R*6 ^simultaneously removes *tub-Gal80*, which leads to disinhibition of Gal4 and, thus, drives the expression of both the *GFP *marker and *Ofut1*^*R*245*A*^.

For genomic rescue experiments, a 3.8 kb BamHI/EcoRI fragment comprising the *Ofut1 *genomic sequence flanked by partial sequences of the neighboring genes *CG8257 *and *CG8309*, was cloned into the corresponding sites of pCasper. The R245A mutation was introduced by site directed mutagenesis (Stratagene). The resulting construct, *pCasper-Ofut1*^*R*245*A*^, was subjected to transposon-mediated germline transformation. To recombine with *Ofut1*^4*R*6^, lines with insertions on the right arm of chromosome 2 were selected. Three lines, *Ofut1*^*R*245*A*^*[18.1] *(insertion; 43C), *Ofut1*^*R*245*A*^*[33.1] *(insertion; 53E) and *Ofut1*^*R*245*A*^*[23.1] *(insertion; 59A-C), are obtained. Among them, the expression was confirmed in *Ofut1*^*R*245*A*^*[18.1] *and *Ofut1*^*R*245*A*^*[23.1]*. As the recombination between *Ofut1*^*R*245*A*^*[18.1] *and *FRT42B [G13] *was difficult owing to the proximity of each insertion site, we used *Ofut1*^*R*245*A*^*[23.1] *for further analysis.

For expression of GFP:KDEL in wing disk cells, the GFP:KDEL coding fragment was isolated by polymerase chain reaction (PCR) from pMT/Bip-GFP:KDEL [10], cloned into pUAST and transformed in to *Drosophila*.

Antibody staining was performed essentially as described previously [5]. Antibodies used were mouse anti-WG (4D4, DSHB), mouse anti-Notch (C458.2H, DSHB), rat anti-ELAV (7E8A10, DSHB), rabbit anti-HRP (Cappel), rabbit anti-Notch (Intracellular Notch, E. Giniger), guinea pig anti-OFUT1 [10], guinea pig anti-Boca antibody [29], mouse anti-KDEL antibody (Stressgen) and rabbit anti-Calnexin antibody (Stressgen).

### Cell surface Notch staining

For cell surface Notch staining of wing disks, third instar larvae were dissected and fixed for 30 min with the fixative solution containing 4% formaldehyde (Polysciences Inc. #18814), 0.1 M Pipes (pH7.2) and 50 mM NaCl. After washing three times for 5 min with phosphate buffered saline (PBS; 10 mM sodium phosphate, 2.7 mM KCl, 137 mM NaCl) followed by blocking with PBS plus 5% donkey serum (PBS-DS) for 30 min, disks were incubated with mouse anti-extracellular Notch (C458.2H; DSHB) in PBS-DS overnight. After washing with PBS (three times for 5 min) followed by blocking with PBS-DS for 30 min, disks were incubated for 3 h with Cy3-conjugated donkey anti-mouse IgG (Jacson Laboratories) in PBS-DS containing 0.05% BSA and 0.005% Triton-X 100, and then washed three times for 5 min with PBS containing 1% BSA and 0.1% Triton-X 100 (PBT).

## List of abbreviations

A-P, anterior-posterior; D-V, dorsal-ventral; FNG, Fringe; GlcNAc, *N*-acetylglucosamine; GMD, GDP-D-mannose dehydratase; MARCM, mosaic analysis with a repressible cell marker; OFUT1, *O*-fucosyltransferase1; PBS, phosphate buffered saline; PBS-DS, PBS plus 5% donkey serum; PCR, polymerase chain reaction; WG, Wingless.

## Authors' contributions

TO performed genetic and histochemical analyses in Figures 1, 2, 3 and 4A–D and Additional file 1F. BVVGR performed genetic and histochemical analyses in Figures 4E and 4F and Additional file 1A–E and 1G. TM provided general assistance in the genetic experiments in Figures 1B, 1C, 2E, 2F and 3A–D. TO and KDI conceived of the study, participated in its design and coordination and helped to draft and edit the manuscripts. All authors read and approved the final manuscript.

## Supplementary Material

Additional file 1 **Distribution of various ER markers in the wing disks**. Vertical section is shown with apical up in all panels. (A) *UAS-iOfut1 [16.2]; ptc-Gal4 *wing disk immunostained with OFUT1 (green) and Calnexin (magenta). This line exhibits only partial silencing of OFUT1 expression, visible on the left-hand side of the panel. (B), (C) Immunostaining of wild-type wing disks with (B) Boca (green) and Calnexin (magenta) or (C) OFUT1 (green) and KDEL (magenta). (D), (E) Wing disks expressing GFP:KDEL (magenta) under *ptc-Gal4 *control are immunostained with (D) Boca or (E) OFUT1. (F), (G) *UAS-iOfut1 [12.3]; ptc-Gal4 *wing disks raised at 29°C are immunostained with Notch (magenta) and (F) KDEL or (G) Boca (green). The dashed line marks the edge of the *ptc *expression stripe. Click here for file
