# Supplementary figures and images for: Contributions of chaperone and glycosyltransferase activities of O-fucosyltransferase 1 to Notch signaling
Source: BMC Biol. 2008 Jan 14;6:1. doi: 10.1186/1741-7007-6-1 (PMC2242781; doi:10.1186/1741-7007-6-1)

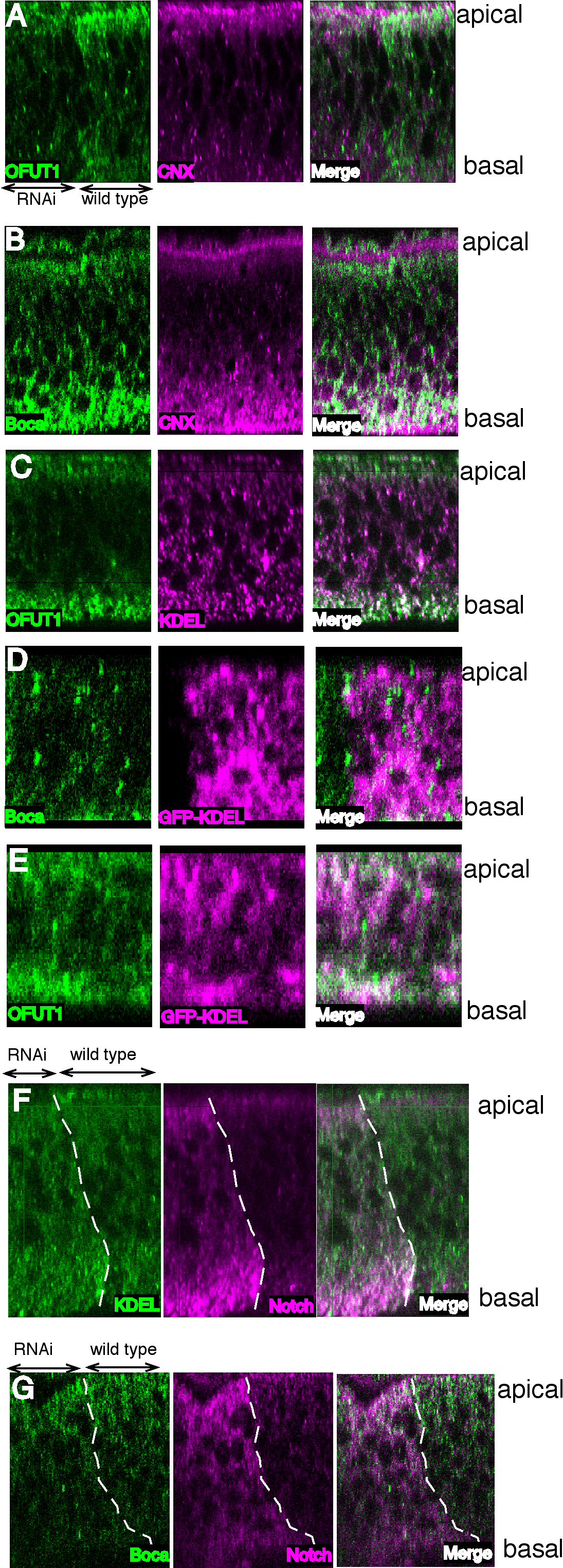

Supplement: Additional file 1 — Distribution of various ER markers in the wing disks. Vertical section is shown with apical up in all panels. (A) UAS-iOfut1 [16.2]; ptc-Gal4 wing disk immunostained with OFUT1 (green) and Calnexin (magenta). This line exhibits only partial silencing of OFUT1 expression, visible on the left-hand side of the panel. (B), (C) Immunostaining of wild-type wing disks with (B) Boca (green) and Calnexin (magenta) or (C) OFUT1 (green) and KDEL (magenta). (D), (E) Wing disks expressing GFP:KDEL (magenta) under ptc-Gal4 control are immunostained with (D) Boca or (E) OFUT1. (F), (G) UAS-iOfut1 [12.3]; ptc-Gal4 wing disks raised at 29°C are immunostained with Notch (magenta) and (F) KDEL or (G) Boca (green). The dashed line marks the edge of the ptc expression stripe. [file 1741-7007-6-1-S1.TIFF]
